# Fine-Tuning of *hemB* Using CRISPRi for Increasing 5-Aminolevulinic Acid Production in *Escherichia coli*

**DOI:** 10.3389/fmicb.2019.01731

**Published:** 2019-07-31

**Authors:** Tianyuan Su, Qi Guo, Yi Zheng, Quanfeng Liang, Qian Wang, Qingsheng Qi

**Affiliations:** ^1^State Key Laboratory of Microbial Technology, National Glycoengineering Research Center, Shandong University, Qingdao, China; ^2^CAS Key Lab of Biobased Materials, Qingdao Institute of Bioenergy and Bioprocess Technology, Chinese Academy of Sciences, Qingdao, China

**Keywords:** fine-tuning, CRISPR interference, 5-aminolevulinic acid, *hemB*, Escherichia coli

## Abstract

5-aminolevulinic acid (5-ALA) is an important metabolic intermediate in the biosynthesis of heme and has been broadly applied in medicine, agriculture, and organic synthesis. Compared to the chemical synthesis methods, microbial fermentation of ALA has significant economic and environmental advantages. However, the heme biosynthesis pathway downstream of ALA is essential for cell survival, so it cannot be completely blocked. In this work, we fine-tuned the expression of HemB, the key enzyme involved in heme biosynthesis, using CRISPR interference (CRISPRi), and investigated its effect on promoting ALA accumulation. The activity of HemB was down-regulated by 15, 19, 33, 36, 71, and 80% respectively, with six CRISPRi sites targeting various regions of *hemB*. ALA accumulation in these *hemB* weakened strains varied from 90.2 to 493.1% compared to that of the original strain. This work provided new insights into fine-tuning of heme biosynthesis pathway for promoting ALA production.

## Introduction

5-aminolevulinic acid (ALA) is a non-protein amino acid involved in heme synthesis and has attracted increasing interest among researchers due to its potential applications in medicine and agriculture (Kang et al., 2012). ALA can be used as photosensitizer for photodynamic diagnosis (PDD) and photodynamic therapy (PDT) in cancer treatment (Peng et al., 1997; Khaled et al., 2015). ALA can also be used as biodegradable herbicide, insecticide, and growth-accelerating agent in agriculture (Sasaki et al., 1987; Kang et al., 2012). Currently, ALA is primarily chemically synthesized from tetrahydrofurfurylamine, furfural, or levulinic acid. Compared with the chemical synthesis method, microbial fermentation of ALA is green, energy-efficient, and economical, which has attracted intensive attention (Nishikawa et al., 1999; Fu et al., 2010; Yu et al., 2015).

**FIGURE 1:**
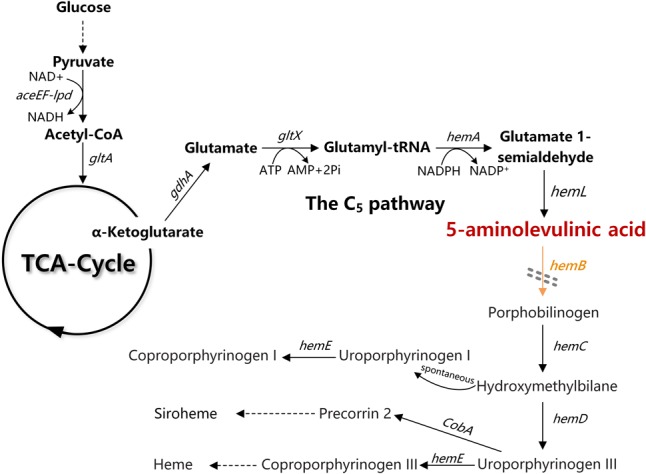
The ALA biosynthesis and degradation pathway in *Escherichia coli.* The gray double-dashed lines indicate the genes that were attenuated. *gdhA*, glutamate dehydrogenase; *gltX*, glutamyl-tRNA synthetase; *hemA*, glutamyl tRNA reductase; *hemL*, glutamate-1-semialdehyde aminotransferase; *hemB*, 5-aminolevulinic acid dehydratase.

In *Escherichia coli*, ALA is natively synthesized via the C5 pathway. ALA biosynthesis through this pathway is tightly regulated by the downstream metabolite heme (Figure 1; Woodard and Dailey, 1995). In C5 pathway, glutamate is first ligated to t-RNA by glutamate-tRNA ligase (GluRS, encoded by *gltX*). The formed glutamyl-tRNA is subsequently reduced to glutamate-1-semialdehyde (GSA) by glutamyl-tRNA reductase (HemA, encoded by *hemA*). Finally, GSA is quickly converted to 5-ALA through the catalysis of glutamate-1-semialdehyde aminotransferase (HemL, encoded by *hemL*). Previously, we have constructed a recombinant *E. coli* for efficient production of ALA from glucose via the C5 pathway. Through heterologous expression of stabilized *hemA* from *Salmonella arizona* and co-expression with *hemL* in *E. coli*, ALA production reached 2052 mg/L in modified minimal medium from glucose (Kang et al., 2011). However, we found that overexpression of *gltX* up-regulated the transcription of *hemB* and reduced the accumulation of ALA. This is consistent with the report that *gltX* and *hemA* are subjected to tightly feedback inhibition by their end product heme (Li et al., 2014). These phenomena suggest that the heme biosynthesis pathway affects the accumulation of ALA.

Heme is the cofactor of many enzymes involved in the election transport chain and is essential for cell survival (Mobius et al., 2010). Completely blocking the heme biosynthesis pathway is impracticable. The ALA dehydratase (HemB, encoded by *hemB*) is responsible for catalyzing the condensation of two ALA molecules into porphobilinogen (PBG), which is the rate-limiting enzyme of ALA catabolism and heme biosynthesis (Figure 1). Many studies showed that down-regulation of the activity of HemB is vital for increasing the ALA production. Sasaki et al. demonstrated the supplement of levulinic acid (LA), an inhibitor of HemB, into the anaerobic-light culture of *Rhodobacter sphaeroides* during the middle log-phase accelerated the formation of extracellular ALA (Sasaki et al., 1987). Later, Lee et al. (2003) showed that the presence of 0.5–10 mM of D-glucose caused a concentration-dependent inhibition of HemB activity in recombinant *E. coli* and increased the accumulation of ALA. D-xylose has also been found as a new inhibitor for HemB, and it is conducive to ALA production (Lin et al., 2009). HemB inhibitors can effectively promote ALA accumulation. However, it is not an ideal solution because it will increase the cost for industrial fermentation. Adding the ssrA, protein degradation tag to *hemB* in *Corynebacterium glutamicum* can accelerate the degradation of HemB (Yu et al., 2015). However, the ALA production did not increase, probably because the specific constant degradation rate conferred by ssrA tag was not suitable for the strain to balance heme metabolism and ALA biosynthesis. Consequently, regulating, not directly blocking, the metabolic flux of ALA into downstream metabolites at a suitable level is a promising strategy for increasing the accumulation of ALA (Leeper, 1989; Frankenberg et al., 2003; Kwon et al., 2003).

The recently developed CRISPR interference (CRISPRi) enables the down-regulation of the specific gene expression with diverse degrees, and it also has been widely applied in tuning the metabolic flux in various microbes (Bikard et al., 2013; Qi et al., 2013). CRISPRi is able to bind specific DNA sequences through a catalytically inactive Cas9 protein (dCas9) and down-regulation the transcription of targeted genes. Chen et al. reported that through the design of the five various sgRNAs targeting the native gene *sad* in *E. coli*, carbon flux to the 4-hydroxybutyrate (4HB) biosynthesis was fine-regulated and allowed various 4HB contents in P(3HB-co-4HB) ranging from 1 to 9 mol% (Lv et al., 2015). Recently, a xylose-induced CRISPRi system was established in *Bacillus subtilis* to down-regulate the expression of three genes (*zwf, pfkA, glmM*) involved in the three major competing reactions of GlcNAc synthesis [pentose phosphate pathway (HMP), glycolysis, and peptidoglycan synthesis pathway (PSP)] (Wu et al., 2018). Simultaneous inhibition of these three genes by CRISPRi increased GlcNAc titer by 13.2% to 17.4 ± 0.47 g/L. Combinatorial inhibition using 27 arrays containing sgRNAs with different repression capacities targeting the three genes obtained the most efficient strain, BNX122, which produced 20.5 ± 0.85 g/L of GlcNAc with a yield of 0.46 ± 0.010 g/g glucose and xylose in shake flask culture. These works demonstrated that CRISPRi is efficient for the fine-tuning of single or multiple genes to a suitable level in the metabolic pathway and promotes the production of value-added compounds.

In this study, the flux of heme biosynthesis was elaborately modulated through fine-tuning the expression of *hemB* using CRISPRi. The activity of HemB was down-regulated by 15, 19, 33, 36, 71, and 80%, respectively. The ALA accumulation of these *hemB*-weakened strains was investigated. This work demonstrated that fine-tuning of heme biosynthesis pathway was able to maintain intracellular heme at the suitable level and to maximize ALA production.

## Materials and Methods

### Bacterial Strains and Plasmids Construction

All bacterial strains, plasmids, and primers used in this work were summarized in Tables 1–3. *E. coli* DH5α and BW25141 were used as the hosts for molecular cloning and manipulation of plasmids. To construct the integrative plasmid, pKTAL, two DNA fragments, R6K-FRT-*kan* and *tac-hemA^M^-hemL* were amplified using RFK-F/RFK-R and HemAL-F/HemAL-R as the primers and plasmids, pG-2 and pDAL as the templates, respectively. The Phanta HS super-fidelity DNA polymerase was purchased from Vazyme Biotech (Nanjing, China). The two DNA fragments were then assembled *in vitro* using Gibson assembly and transferred into *E. coli* strain BW25141 for further replication and verification.

**TABLE 1:** Strains used in this study.

**Strains**	Genotype	**Source**
DH5α	F^–^, endA1, hsdR17 (rK^–^, mK^+^), supE44, thi-l, λ^–^, recA1, gyrA96,ΔlacU169 (Φ80lacZΔM15)	Lab stock
MG1655	Wild type	Lab stock
BW25141	F-, Δ(araD-araB)567, ΔlacZ4787(::rrnB-3), Δ(phoB-phoR)580, λ-, galU95, ΔuidA3::pir+, recA1, endA9(del-ins)::FRT, rph-1, Δ(rhaD-rhaB)568, hsdR514	Lab stock
DH-2	*E. coli* DH5α (Δ*arcA*::FRT, Δ*recA*::FRT)	This work
DHAL	DH-2 containing seven copies of *hemA*^*M*^ and *hemL* on the chromosome	This work
DH-GFP	*E. coli* MG1655 (Δ*ptsG*::*kan-trc-gfp*)	This work
DH-crRNA	DH-GFP containing pdCas9	This work
DH-B1	DH-GFP containing pdCas9-B1	This work
DH-B2	DH-GFP containing pdCas9-B2	This work
DH-B3	DH-GFP containing pdCas9-B3	This work
DH-T1	DH-GFP containing pdCas9-T1	This work
DH-T3	DH-GFP containing pdCas9-T3	This work
DH-T4	DH-GFP containing pdCas9-T4	This work
DH-T6	DH-GFP containing pdCas9-T6	This work
DH-T10	DH-GFP containing pdCas9-T10	This work
DHAL-crRNA	DHAL containing pdCas9	This work
DHAL-H4	DHAL containing pdCas9-H4	This work
DHAL-H5	DHAL containing pdCas9-H5	This work
DHAL-H10	DHAL containing pdCas9-H10	This work
DHAL-H12	DHAL containing pdCas9-H12	This work
DHAL-HR5	DHAL containing pdCas9-HR5	This work
DHAL-HR6	DHAL containing pdCas9-HR6	This work

**TABLE 2:** Plasmids used in this study.

**Plasmids**	Relevant genotype	**References**
pKTAL	*oriR6Kγ*, FRT-*kan-tac-hemA^M^-hemL*, Kan^*R*^	This work
pG-2	*oriR6Kγ*, FRT-*kan*-*trc*-*gfp*, Kan^*R*^	Gu et al., 2015
PDAL	pUC19 containing *hemA*^*M*^ (*S. arizona*) and *hem L* (*E. coli*), Amp^*R*^	Kang et al., 2011
pKD3	FRT-*kan*-FRT, Amp^*R*^	Datsenko and Wanner, 2000
pTKRED	temperature-conditional replicon containing γ, *β, exo* (red recombinase), Spc^*R*^	Kuhlman and Cox, 2010
pCP20	helper plasmid, Cm^*R*^	Cherepanov and Wackernagel, 1995
pLYK	pCL1920 containing FRT-*kan*-*trc*-*gfp* operon, Spc^*R*^	Gu et al., 2015
pdCas9	pACYC184 containing tracr RNA, *dCas9* and CRISPR array, Cm^*R*^	Bikard et al., 2013
pdCas9-B1	pdCas9 containing target B1	This work
pdCas9-B2	pdCas9 containing target B2	This work
pdCas9-B3	pdCas9 containing target B3	This work
pdCas9-T1	pdCas9 containing target T1	This work
pdCas9-T3	pdCas9 containing target T3	This work
pdCas9-T4	pdCas9 containing target T4	This work
pdCas9-T6	pdCas9 containing target T6	This work
pdCas9-T10	pdCas9 containing target T10	This work
pdCas9-H4	pdCas9 containing target H4	This work
pdCas9-H5	pdCas9 containing target H5	This work
pdCas9-H10	pdCas9 containing target H10	This work
pdCas9-H12	pdCas9 containing target H12	This work
pdCas9-HR5	pdCas9 containing target HR5	This work
pdCas9-HR6	pdCas9 containing target HR6	This work

**TABLE 3:** Primers used in this study.

**Primers**	**Nucleotide sequence (5′-3′)**
RFK-F	CTGGCGTTATCTGGTAAGGTTGGGAAGCCCTGCCATGTCAGCCGTTAAGTGTTCCT
RFK-R	ACACATTATACGAGCCGATGATTAATTGTCAATCAGAAGAACTCGTCAAGAAGGCG
HemAL-F	TTGACAATTAATCATCGGCTCGTATAATGTGTGGAATTGTGTGTGGAATTGTGAGCGGATAACAAT
HemAL-R	CAGGGCTTCCCAACCTTACCAGATAACGCCAGGGTTTTCCCAGTCACG
gfp-F	GCACCCATACTCAGGAGCACTCTCAATTATGTTTAAGAATGCATTTGGTGAATTCGAGCTCGGTACCCGGGGATC
gfp-R	CAGCCATCTGGCTGCCTTAGTCTCCCCAACGTCTTACGGATTACAGGAAACAGCTATGACCATGATTAC
arcA-F	ACTTCCTGTTTCGATTTAGTTGGCAATTTAGGTAGCAAACAGTGTAGGCTGGAGCTGCTTC
arcA-R	TTAATCTTCCAGATCACCGCAGAAGCGATAACCTTCACCGTGAATGGGAATTAGCCATGGTCC
recA-F	ATTGACTATCCGGTATTACCCGGCATGACAGGAGTAAAAGTGTAGGCTGGAGCTGCTTC
recA-R	AGGGCCGCAGATGCGACCCTTGTGTATCAAACAAGACGAATGGGAATTAGCCATGGTCC
gfp-testF	GATGCCCTGTACACGGCGAGGCTCT
gfp-testR	CACGTATCAATTCTGAATAACACC
arcA-testF	ACGCAATTACGTACTTTAGTCATG
arcA-testR	ACGGACGATGAGTTACGTATCTG
recA-testF	TATGCATTGCAGACCTTGTGGCAACAA
recA-testR	CACGTAAGAGGTTCCAACTTTCAC
**Primers for RT-PCR**	
Kan-RT-F	CTGCTATTGGGCGAAGTG
Kan-RT-R	CTGCTATTGGGCGAAGTG
gapART-F	AACTGAATGGCAAACTGACTGGTA
gapART-R	TTTCATTTCGCCTTCAGCAGC
gltX-RT-F	TGAAAGAGATGGCACAGAGC
gltX-RT–R	GCGGTCCAGTCAGTAATCG
*hemA*-RT-F	CCAGGCAGAGCAAGTTCG
*hemA*-RT-R	ATTCAGGCGTTCGTTATCCC
*hemL*-RT-F	TGGTCGTCGTGATGTAATGG
*hemL*-RT-R	GCTTCTTCTGCCGCTTCC
*hemB*-RT-F	TGGGCTTCATCCAGCAGTGA
*hemB*-RT-R	GGCGATTATGTCGTATTCGACCA
*hemC*-RT-F	GTCGGGACGTCCAGTTTACG
*hemC*-RT-R	GCTACGGCAAGAATGATGGC
*hemD*-RT-F	TATCGTGAGCACTGGTTACTAC
*hemD*-RT-R	CATCGTTGTCAGCGTTATCG
*hemE*-RT-F	GTTACGTGATGAACGCGGTG
*hemE*-RT-R	GATCACGGTGAAGGCTTTGC

Plasmid pdCas9 was gifted from Luciano Marraffini (Addgene plasmid # 4656), which introduced D10A and H840A mutations in Cas9 for abolishing the cleavage activity. The new spacer sequences were inserted into pdCas9 via the Golden Gate assembly as previously reported. Briefly, pdCas9 was digested with *Bsa*I and then ligated with the annealed complementary oligonucleotides containing corresponding sticky ends. Complementary oligonucleotides used in the study are listed in Table 4.

**TABLE 4:** Complementary oligonucleotides used in this study.

**Oligos**	**Nucleotide sequence (5′-3′)**
B1-F	AAACCTCTTTCTCTAGACCACACATTATACGAGC
B1-R	AAAAGCTCGTATAATGTGTGGTCTAGAGAAAGAG
B2-F	AAACTTAACATCACCATCTAATTCAACAAGAATT
B2-R	AAAAAATTCTTGTTGAATTAGATGGTGATGTTAA
B3-F	AAACGTAGTTTTCCAGTAGTGCAAATAAATTTAA
B3-R	AAAATTAAATTTATTTGCACTACTGGAAAACTAC
T1-F	AAACTCTGAAATGAGCTGTTGACAATTAATCATC
T1-R	AAAAGATGATTAATTGTCAACAGCTCATTTCAGA
T3-F	AAACCGGCTCGTATAATGTGTGGTCTAGAGAAAG
T3-R	AAAACTTTCTCTAGACCACACATTATACGAGCCG
T4-F	AAACAGAGAAAGAGGAGAAATACTAGATGAGTAA
T4-R	AAAATTACTCATCTAGTATTTCTCCTCTTTCTCT
T6-F	AAACATTCTTGTTGAATTAGATGGTGATGTTAAT
T6-R	AAAAATTAACATCACCATCTAATTCAACAAGAAT
T10-F	AAACTCAAGAGTGCCATGCCCGAAGGTTATGTAC
T10-R	AAAAGTACATAACCTTCGGGCATGGCACTCTTGA
H4-F	AAACTGTTGACAAGAAGAGAATGGAAGAGAGGCCG
H4-R	AAAACGGCCTCTCTTCCATTCTCTTCTTGTCAACA
H5-F	AAACCGAGGGGCATAGTATACCTGAAGCAGGGTAG
H5-R	AAAACTACCCTGCTTCAGGTATACTATGCCCCTCG
H10-F	AAACAACATAGCGCGCAGCGCAGGAGATTTGCGCG
H10-R	AAAACGCGCAAATCTCCTGCGCTGCGCGCTATGTT
H12-F	AAACGAATGCGCATCACGCCTGGCATGGCTTCAAG
H12-R	AAAACTTGAAGCCATGCCAGGCGTGATGCGCATTC
HR5-F	AAACGCAGCGATGCCTGGCGGGAAGATGGACTGGG
HR5-R	AAAACCCAGTCCATCTTCCCGCCAGGCATCGCTGC
HR6-F	AAACAGACACCTGCTTCTGTGAATACACTTCTCAG
HR6-R	AAAACTGAGAAGTGTATTCACAGAAGCAGGTGTCT

### Gene Deletion and Insertion

To generate DH-2, *recA* and *arcA* were knocked out in *E. coli* strain DH5α using one-step inactivation method (Datsenko and Wanner, 2000). Linearized DNA fragments corresponding to the homologous sequences were amplified via PCR using pKD3 as template. After induction of the expression of λ-Red recombinases from pTKRED, the PCR products were electrotransformed into the *E. coli* cells for recombineering. Colonies formed on the antibiotic plates were identified by colony PCR using 2xTaq Mastermix (Cwbio, China).

To generate DHAL, the strain DH-2 containing pCP20 overexpressing the FLP recombinase was transferred into 42°C for 20–30 min to induce the expression of FLP recombinase. The integrative plasmid, pKTAL, was then electrotransformed into the host strain and was screened on the LB agar plate with kanamycin.

To generate DH-GFP, the *kan*-*trc*-*gfp* cassette was inserted into the *E. coli* MG1655 strain using the one-step inactivation method as previously reported (Datsenko and Wanner, 2000). The *kan*-*trc*-*gfp* cassette was amplified from pLYK by the primers, gfp-F/gfp-R.

### Growth Conditions

Strains for cloning and inoculums were grown in Luria-Bertani media (10 g /L tryptone, 5 g/L yeast extract, and 10 g/L NaCl) supplemented with appropriate antibiotics (100 mg/L ampicillin; 25 mg/L kanamycin; 25 mg/L chloramphenicol, and 50 mg/L spectinomycin) at 37°C or 30°C as indicated for 8 to 12 h. The modified minimal medium containing 16 g/L (NH_4_)_2_SO_4_, 3 g/L KH_2_PO_4_, 16 g/L Na_2_HPO_4_⋅12H_2_O, 1 g/L MgSO_4_⋅7H_2_O, 0.01 g/L MnSO_4_⋅7H_2_O and 2 g/L yeast extract was used for fermentation. Glucose as sole carbon source was added as indicated.

For flask cultivation, strains were first cultured in 5 mL of Luria-Bertani medium at 37°C. The overnight cultures (1 mL) were subsequently inoculated into 50 mL of Luria-Bertani medium, cultured for 8 to 12 h, and then 5% (v/v) seed cultures for batch cultivation were incubated into 50 mL modified minimal medium in 300 mL baffled Erlenmeyer flask at 37°C with 220 rpm. As the sole carbon source, 20 g/L of glucose was added initially.

For fed-batch fermentation, a stirred 5-L glass vessel with BioFlo310 modular fermentor system (New Brunswick Scientific, United States) containing 3.5 L modified minimal medium was used. The inoculum ratio was 5% (v/v), and glucose was initially added at a concentration of 35 g/L. When glucose concentration went below 10 g/L, a feeding solution containing 500 g/L of glucose was added to the medium. The incubation temperature was controlled at 37°C, and the dissolved oxygen concentration was maintained at 30% through adjusting the agitation speed and aeration rate. The pH was controlled at 6.5 ± 0.2 with 5 M NaOH.

### Quantitative Real-Time PCR (RT-PCR) Analysis

The integrated copy number of *hemA* and *hemL* was determined by RT-PCR. The genome DNA was isolated from the candidate strains using TIANamp Bacteria DNA Kit (Tiangen Biotech, Beijing, China). *gapA* encoding D-glyceraldehyde-3-phosphate dehydrogenase was used as internal standard. RT-PCR was performed using SYBR Premix Ex TaqII kit (Takara Bio, Dalian, China) with a LightCycler 480 according to the manufacturer’s instructions. To measure the mRNA expression of target genes in the *hemB*-weakened strains, the total RNAs of various *hemB*-weakened strains were extracted from the 20 h fermentation broth using the Bacterial RNA Kit (Omega Bio-Tek, Doraville, GE, United States). Reverse transcription was performed with the total RNAs as the templates using the ReverTra Ace qPCR RT Master Mix with gDNA Remover (Toyobo, Shanghai, China). Gene expression analysis was carried out in a 96-well plate with a total reaction volume of approximately 25 μL using a SYBR Green Realtime PCR Master Mix (Toyobo, Shanghai, China). The relative transcription level of the target genes was quantified by the 2^–ΔΔ*CT*^ method using the *gapA* gene as internal control. For each sample, three repetitions were performed. Primers used in RT-PCR are listed in Table 3.

### Analytical Method

Optical density at 600 nm (OD_600_) was measured using the spectrophotometer (Shimadzu, Japan). Glucose and glutamate were analyzed using a SBA-40C biosensor (Biology Institute of Shandong Academy of Sciences). The fluorescence intensity of green fluorescence protein was determined in 96-well microtiter plates with excitation at 485 nm and emission at 528 nm using a Multi-Detection Microplate Reader (BioTek, United States). The crude enzyme activity of HemB was quantified using the method described previously (Sassa, 1982). ALA concentration was analyzed using modified Ehrlich’s reagent as previously described (Mauzerall and Granick, 1956).

## Results

### Fine-Tuning the Expression of GFP Using CRISPRi

**FIGURE 2:**
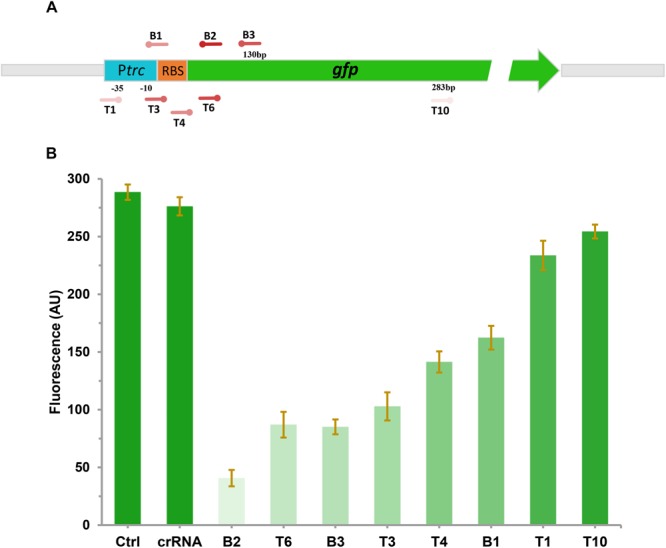
Repression of green fluorescent protein (GFP) by CRISPRi. **(A)** The distribution of CRISPRi targets on the *gfp* operon. The deeper the color of the target sites represents the stronger the repression of GFP by CRISPRi. **(B)** The fluorescent intensity of DH-GFP containing different CRISPRi targets. The *error bars* indicate standard deviations from three independent measurements.

To investigate the effect of CRISPRi on repressing the transcription of targeted gene with different levels, a constitutive expressed GFP report system was constructed. The *gfp* gene under the control of *trc* promoter was integrated into the chromosome of *E. coli* DH5α (generating DH-GFP) for stable expression of GFP. To explore the inhibitory effect of GFP with various CRISPR target sites, eight spacers sites were designed on the *gfp* gene. T1, T3, and B1 were located on the promoter or RBS region in front of *gfp* (Figure 2A). B2 and B3 were distributed in the coding chain of *gfp*, the remaining (T4, T6, T10) were distributed in the non-coding chain (Figure 2A). After transferring these target sites into the *gfp* expression strain, DH-GFP, the intensity of the green fluorescence decreased to various degrees, ranged from 8 to 85% (Figure 2B). The most effective site, B2, was located on the front region of the coding chain (65 bp from the start codon). T6 targeting the same region of the non-coding chain as B2 exhibited slightly lower inhibition of GFP (73.7 vs. 85%). B3 targeting the coding chain behind B2 repressed 54% of the green fluorescence expression relative to the wild type strain. The remaining sites T1, T3, and B1 targeting the promoter or RBS region of *gfp* achieved 13, 62, and 47% fluorescence inhibition, respectively. These indicated that the expression of *gfp* could be modulated by CRISPRi, and a broadly regulating range could be achieved through designing various CRISPRi target sites.

### Construction of a Stably Expressed C5 ALA Synthetic Pathway in *E. coli*

**FIGURE 3:**
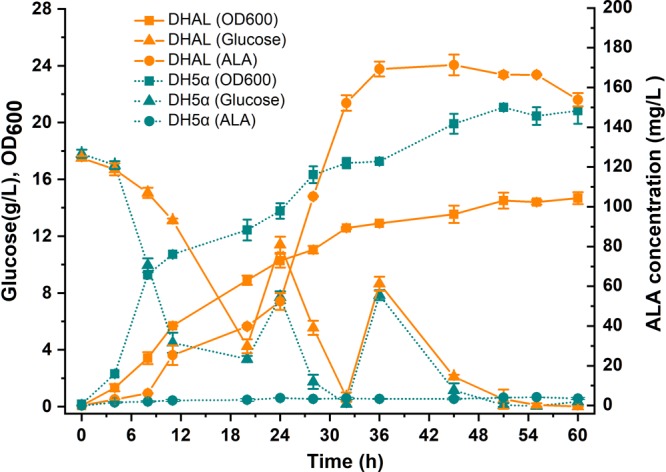
Batch cultivation of *E. coli* DH5α and DHAL in 300 mL shake flasks. The results are the average of three individual experiments.

Since down-regulation of the heme biosynthesis pathway decreased the microaerobic tolerance of the host cell, the *arcA* gene was first knock-out in *E. coli* strain DH5α. ArcA is a global transcription regulator that affects the activity of many aerobic function enzymes in the anoxic growth condition (Malpica et al., 2006; Li et al., 2014). To construct the stabilized ALA production strain, the ALA C5 biosynthesis pathway (*hemA*^*M*^ from *Salmonella arizona* and *hemL* from *E. coli*) was integrated into the host chromosome using the chromosomal integration of gene(s) with multiple copies (CIGMC) method (Gu et al., 2015). The final strain DHAL was integrated with seven copies of *hemA*^*M*^ and *hemL*, identified by RT-PCR. ALA production in batch fermentation was 169.6 mg/L, and the fermentation broth in dark red suggested that excessive heme was accumulated in the resulting strain (Figure 3). In addition, the biomass of DHAL was lower than the parental strain, further indicating that the excess of heme disturbed the normal cellular respiration and suppressed cell growth (Figure 3).

### Down-Regulation of *hemB* at Different Levels Using CRISPRi

**FIGURE 4:**
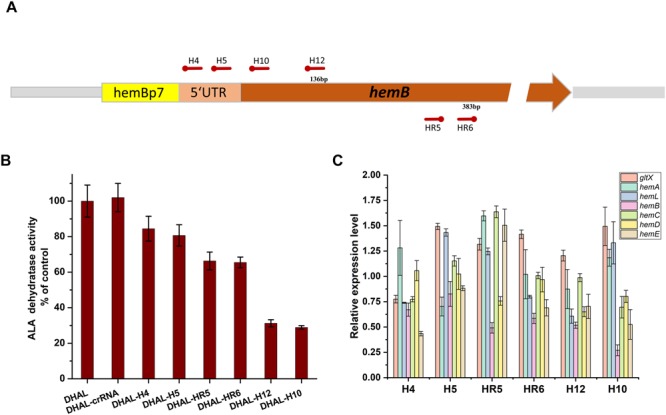
Down-regulation of *hemB* by CRISPRi. **(A)** The distribution of CRISPRi targets on the *hemB* operon. **(B)** Enzyme activity of ALA dehydratase in DHAL containing different CRISPRi targets. The *error bars* indicate standard deviations from three independent measurements. **(C)** Relative gene expression in DHAL containing different CRISPRi targets. The control was DHAL harboring pdCas9. The transcription level of the target gene was quantified by the 2^–ΔΔ*CT*^ method using the *gapA* gene as the internal control. The *error bars* represent standard deviations from three independent experiments.

To balance heme metabolism and ALA biosynthesis in the ALA production strain DHAL, the ALA dehydratase encoded by *hemB* was down-regulated using CRISPRi. Six specific spacers targeting different regions of *hemB* were designed to repress the activity of HemB at various levels (Figure 4A). In order to obtain a broad repression range, H4 and H5 were designed on the RBS region of *hemB*, H10 and H12 in the front region of *hemB*, targeting the coding chain, which has been demonstrated as an efficient down-regulation of the expression of *gfp* region, while the other two spacers, HR5 and HR6, were targeting the non-coding chain of *hemB*, which may cause weaker repression than targeting the coding DNA chain (Bikard et al., 2013).

The activity of HemB was quantified to determine the inhibition efficiency of *hemB* (Figure 4B). A broad down-regulating range of HemB, from 15.5 to 71.1%, was achieved with various CRISPRi sites. Among them, H10 targeting the coding strand of *hemB*, distanced 32 bp from the start codon, achieved the strongest repression of HemB. H12, targeting the same strand but slightly farther from the start codon (132 bp from the start codon), also repressed the activity of HemB, effectively. However, H4 and H5 targeting the RBS region of *hemB* only inhibited 15.5 and 19.3% of HemB activity, respectively. The activity of HemB with the remaining two spacers, HR5 and HR6, targeting the non-coding chain of *hemB* was reduced to 66.3 and 65.5%, respectively.

### Effects of *hemB* Down-Regulation on the Heme Biosynthesis Pathway

To explore the effects of down-regulation of *hemB* on the heme biosynthesis pathway, the transcription levels of genes in heme biosynthesis pathway were determined by RT-PCR (Figure 4C). As expected, the mRNA expression of *hemB* decreased in all the *hemB*-weakened strains and the transcription level of *hemB* among the strains with different CRISPRi target sites varied from 0.27 to 0.82 relative to the control (Figure 4C). Strain H10 had the lowest mRNA expression of *hemB*, which was consistent with the results of HemB activity assay. Moreover, inhibition of *hemB* with CRISPRi target sites up-regulated the mRNA transcription of *gltX* in almost all strains except H4. While the expression of downstream genes *hemC, hemD*, and *hemE* in the *hemB*-weakened strain H10 appeared significant for down-regulation (Figure 4C). These results suggested that down-regulation of *hemB* reduced the metabolic flux from ALA to heme and alleviated the inhibition of ALA synthesis pathway by excessive downstream porphyrins.

### Effect of *hemB*-Weakened Strains on ALA Accumulation

**FIGURE 5:**
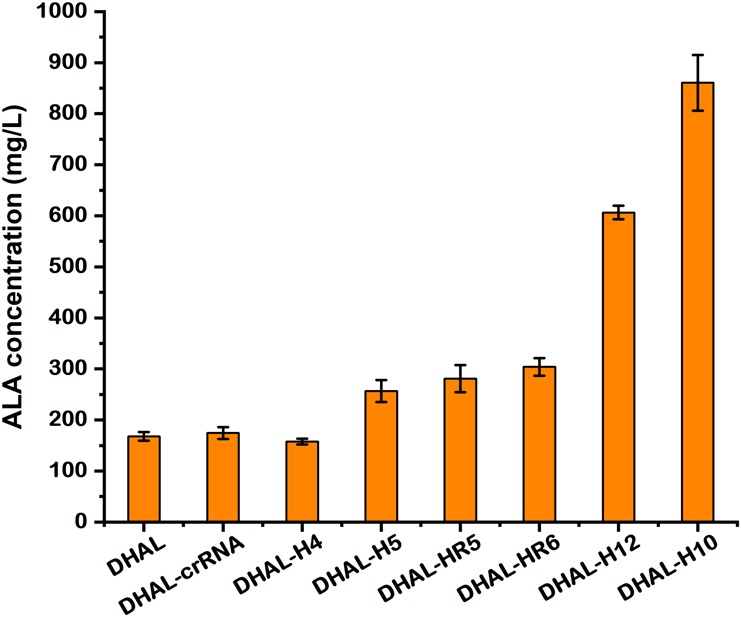
The production of ALA in DHAL containing different CRISPRi targets. The results are the average of three individual experiments.

To determine the capability of ALA accumulation in these *hemB*-weakened strains, batch fermentation was performed. The ALA concentration in DHAL-H10 and DHAL-H12 with strong repression of HemB reached up to 862.0 and 606.3 mg/L (Figures 4B, 5), 5.0-fold and 3.5-fold of the parental strain DHAL, respectively. Additionally, ALA production in DHAL-H4, which had the lesser HemB activity reduction, had no significant change in ALA yield relative to DHAL. The ALA concentration in the remaining three HemB moderate inhibition strains DHAL-H5, DHAL-HR5, and DHAL-HR6 was 256.6, 280.7, and 303.9 mg/L, respectively. We found that ALA production is related to the HemB activity, which makes the higher inhibition of *hemB*, the higher the ALA accumulation (Figures 4, 5).

Furthermore, the accumulation of glutamate was significantly reduced in the *hemB*-weakened strains (Table 5). Among all strains, DHAL-crRNA accumulated 5.42 g/L of glutamate, indicating that excessive ALA resulted in metabolic overflow. However, *hemB*-weakened strain, DHAL-H10 only accumulated 1.81 g/L of glutamate. The color of fermentation broth in the *hemB*-weakened strains also indicated that down-regulation of *hemB* reduced the excessive accumulation of reddish-brown porphyrin compounds in the heme synthesis pathway. As the fermentation time increased, the fermentation broth of the original ALA product strain DHAL gradually turned dusky red. However, the final fermentation broth of DHAL-H10 with the strongest repression of HemB was quite clear. These phenomena further demonstrated that the attenuation of *hemB* expression via CRISPRi reduced the carbon flux from ALA into the downstream heme biosynthesis pathway.

**TABLE 5:** Glutamate accumulation of DHAL containing different CRISPRi targets.

**Strains**	**DHAL**	**DHAL-crRNA**	**DHAL-H4**	**DHAL-H5**	**DHAL-H10**	**DHAL-H12**	**DHAL-HR5**	**DHAL-HR6**
Maximum OD_600_	14.50 ± 1.21	17.04 ± 0.81	19.29 ± 1.35	18.27 ± 0.49	15.41 ± 0.32	11.54 ± 0.81	18.32 ± 1.19	18.17 ± 0.87
Glucose consumption (g/L)	27.62 ± 1.05	35.45 ± 0.32	36.51 ± 0.84	35.10 ± 0.25	36.58 ± 0.56	33.72 ± 0.59	37.11 ± 1.85	37.24 ± 0.93
Glutamate (g/L)	3.8 ± 0.24	5.42 ± 1.23	3.7 ± 0.15	4.8 ± 0.83	1.81 ± 0.12	1.9 ± 0.08	2.6 ± 0.27	2.3 ± 0.33

*Data shown were measured from the samples taken at the time ALA reached to the maximum. Each data indicated the average value of three independent experiments.*

### Fed-Batch Fermentation of DHAL-H10

**FIGURE 6:**
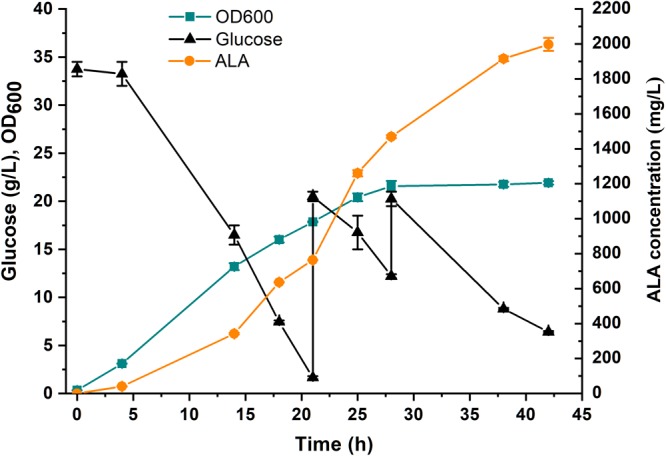
Fed-batch fermentation of DHAL-H10 in 5-L fermentator. A 5% (v/v) inoculum from an overnight culture for 12 h was used. 35 g/L glucose and 0.2 mM IPTG were added initially. During fermentation, the pH was controlled at 6.5 ± 0.5 with 5 M NaOH. The *error bars* indicate standard deviations from three independent measurements.

To investigate the potential of the ALA production in the *E. coli* strain DHAL-H10, fed-batch fermentation in 5-L bioreactor was performed (Figure 6). In the first 28 h, the strains grew exponentially with a rapidly increased ALA accumulation, and the maximum biomass appeared at 28 h. After the cells entered the stationary phase, the cells accumulated ALA slowly, and the finally maximum ALA production reached 1997 mg/L at 42 h. The fermentation process implied that the ALA production was growth-dependent and proved that the fine-tuning of the downstream metabolic flux greatly promoted the accumulation of ALA.

## Discussion

Fine-regulation of the expression of specific genes rather than direct knockout is extremely important for metabolic engineering, especially for genes that affect the cell growth (Na et al., 2013). In the ALA biosynthesis strain, heme is essential for cell survival. Therefore, the carbon flux from ALA to downstream heme biosynthesis cannot be completely blocked. In this study, CRISPRi was applied to fine tune the expression of *hemB* for reducing the metabolic flux from ALA to heme and promoting the accumulation of ALA. CRISPRi can down-regulate gene expression to varying degrees and is therefore widely used in the metabolic engineering for producing various products (Lv et al., 2015; Wu et al., 2015). It has been reported that the fine-regulated expression of *sad* using CRISPRi with five various sgRNAs is able to regulate the carbon flux from glucose to the 4-hydroxybutyrate (4HB) biosynthesis and controls the 4HB content in P(3HB-co-4HB) ranging from 1 to 9 mol% (Lv et al., 2015). In our study, we have further demonstrated that CRISPRi sites targeting different regions on *gfp* can inhibit the expression of *gfp* to varying degrees. The degree of gene down-regulation is correlated with the distance between the CRISPRi target site and the transcription start site (TSS). This powerful ability of CRISPRi provided us a platform to fine-tuning the activity of *hemB*, the essential gene involved heme biosynthesis, and to balance the cell growth and ALA production. As a proof of concept, the yield of the ALA stable strain DHAL with seven copies genome-integrated *hemA^M^L* was increased from 173.6 to 862 mg/L in the best *hemB* weakened strains, demonstrating the feasibility of CRISPRi to fine-regulate *hemB* via selecting suitable CRISPRi site. This is the first time to fine-regulate the ALA biosynthesis pathway and promote ALA production using CRISPRi.

Compared to adding competitive inhibitors directly into the medium to inhibit the activity of HemB, our strategy has two advantages (Sasaki et al., 1987; Saikeur et al., 2009). Firstly, the activity of HemB can be fine-regulated to different degrees using various CRISPRi sites. Therefore, we were able to find the most suitable expression level of *hemB* and achieve up to a three-fold increase in ALA yield. Secondly, regulating heme biosynthesis pathway using CRISPRi makes avoidance of the use of exogenous competitive inhibitors possible, which reduces fermentation costs and simplifies the fermentation process.

In fact, the pathway for both ALA catabolism and heme biosynthesis is quite complex, and the entire regulatory mechanism of these pathways is still unknown (Kwon et al., 2003; Kang et al., 2012). We have previously demonstrated that over-expression of small regulatory RNA, *ryhB*, was able to inhibit the heme synthesis and accelerated ALA accumulation in *E. coli* through a global regulatory mechanism (Li et al., 2014). Zhang et al. (2015) reported that overexpression of certain genes involved in heme biosynthesis pathway, such as *hemD*, and *hemF*, was beneficial to ALA production, whereas, overexpression of *hemB, hemG*, and *hemH* was poisonous to ALA accumulation (Zhang et al., 2015). In this study, we found that the down-regulation of *hemB* reduced the metabolic flux of ALA to downstream porphyrins and therefore attenuated the negative feedback inhibition of ALA synthesis pathway. The mitigation of glutamate overflow during ALA production also demonstrated that down-regulation of *hemB* allowed the ALA biosynthesis pathway more effective. This optimized metabolic pathway was more conducive to ALA production. Although the ALA accumulation has not reached the highest level reported so far, this work provided a new insight into fine-tuning of heme biosynthesis pathway and promoting the production of ALA. By combinatorial regulating of multiple genes involved in heme pathway, it is possibly understand the mechanism of the complicated regulation of heme metabolism and to promote the production of ALA.

## Data Availability

All datasets generated for this study are included in the manuscript.

## Author Contributions

QQ and QW conceived the study. TS, QG, and YZ performed the experiments. TS, QL, QW, and QQ wrote the manuscript. All authors contributed to refining the text and approved the final manuscript.

## Conflict of Interest Statement

The authors declare that the research was conducted in the absence of any commercial or financial relationships that could be construed as a potential conflict of interest.
